# Forecasting the combined effects of future climate and land use change on the suitable habitat of *Davidia involucrata* Baill

**DOI:** 10.1002/ece3.9023

**Published:** 2022-06-17

**Authors:** Junfeng Tang, Xuzhe Zhao

**Affiliations:** ^1^ Key Laboratory of Southwest China Wildlife Resources Conservation (Ministry of Education) China West Normal University Nanchong China

**Keywords:** climate change, Davidia involucrate, land use change, range shifts, SDMs

## Abstract

Accurately predicting the future distribution of species is crucial for understanding how species will response to global environmental change and for evaluating the effectiveness of current protected areas (PAs). Here, we assessed the effect of climate and land use change on the projected suitable habitats of *Davidia involucrata* Baill under different future scenarios using the following two types of models: (a) only climate covariates (climate SDMs) and (b) climate and land use covariates (full SDMs). We found that full SDMs perform significantly better than climate SDMs in terms of both AUC (*p* < .001) and TSS (*p* < .001) and also projected more suitable habitat than climate SDMs both in the whole study area and in its current suitable range, although *D. involucrate* is predicted to loss at least 26.96% of its suitable area under all future scenarios. Similarly, we found that these range contractions projected by climate SDMs would negate the effectiveness of current PAs to a greater extent relative to full SDMs. These results suggest that although *D. involucrate* is extremely vulnerability to future climate change, conservation intervention to manage habitat may be an effective option to offset some of the negative effects of a changing climate on *D. involucrate* and can improve the effectiveness of current PAs. Overall, this study highlights the necessity of integrating climate and land use change to project the future distribution of species.

## Graphical abstract

Future land use patterns would offset some of the negative effects of a changing climate on *D. involucrate*.

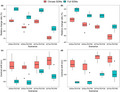

## 1 INTRODUCTION

Global environmental change continues to pose various serious threats to biodiversity and associated ecosystem functioning and services (Bradshaw et al., 2021; Ceballos et al., 2015; Sanderson et al., 2002). In the face of these threats, immediate and thorough conservation efforts are urgently needed (Barnosky et al., 2011), which requires plausible scenarios of expected biodiversity changes under future environmental conditions (Larigauderie et al., 2012; Pereira et al., 2010; Sala et al., 2000), for this forward‐looking approach is essential because drivers of biodiversity changes and their associated impacts change over time (Titeux et al., 2016). Thus, accurately predicting the future distribution of species is crucial for understanding how species will response to global environmental change and has important implications for guiding future conservation planning.

Currently, it has been evidenced that global climate change and land use change are two of most important contemporary factors in driving species redistribution (Newbold, 2018; Powers & Jetz, 2019). However, previous studies focused on modeling spatially explicit patterns of species' range shifts under future environmental conditions by using species distribution models (SDMs) (Dawson et al., 2011; Pearson & Dawson, 2003), a powerful tool for projecting future species ranges (Elith & Leathwick, 2009), have so far mostly considered single factors, focusing extensively on climate change and neglecting other important pressures, such as land use change (Titeux et al., 2016). This approach of single factor to estimate future species ranges may lead to unreliable projections (Marshall et al., 2018; Sirami et al., 2017; Titeux et al., 2017) and has recently been increasing questioned (Marshall et al., 2018; Regos et al., 2016). In this context, several studies have attempted to project species future distribution by integrating climate change and other drivers, such as land‐use change (Elsen et al., 2020; Pant et al., 2021; Zhang et al., 2017) and habitat connectivity (Brun et al., 2020; Huang et al., 2020; Yesuf et al., 2021). This type of integrated approach largely contributes to a deeper understanding of how interactions among drivers may affect the future distribution of species and accordingly is essential to set effective conservation policy and management (Di Febbraro et al., 2019; Zamora‐Gutierrez et al., 2018), and thus has been widely recommended to modeling species future distribution (Titeux et al., 2016).

As a tertiary relict plant endemic to China (Fu & Jin, 1992), the *Davidia involucrata* Baill. currently ranges approximately from 98 to 110°E, 26 to 32°N in southwestern and south‐central China (Li, 1954; Liu et al., 2019; Takhtajan, 1980; Tang et al., 2017). It offers an especially rich system for exploring the interactions of climate change and land use change for several reasons: (a) as a rare species listed in the China Plant Red Data Book under first‐grade state protection, as well as an “Endangered” species on the IUCN Red List of Threatened Species, it is important ecologically and economically (Liu et al., 2019); (b) long‐term national observation records of *D. involucrate* are available in China (Tang et al., 2017; Wang et al., 2019); (c) previous studies have explored the vulnerability of *D. involucrate* to future climate change (Long et al., 2021b; Tang et al., 2017; Wang et al., 2019); (d) despite its populations are often found in subtropical evergreen broad‐leaved forests or in mixed forests of temperate deciduous broad‐leaved trees at high altitudes of between 1100 and 2600 m (He et al., 2004), land use change has led to a sharp decrease of its remaining habitats (Wang et al., 2019); and (e) future climate and land use data under different scenarios are available in China (Fick & Hijmans, 2017; Li et al., 2016).

Here, we aim to (a) examine the combined impacts of climate and land use change on the future distributions of *D. involucrate* and (b) evaluate the effectiveness of current PA networks in protecting *D. involucrate* under the combination of climate change and land use change scenarios. To do so, we compared projected distributions produced by two types of models: (i) including only climate variables (climate SDMs) and (ii) adding also land use variables (full SDMs). We expect that the inclusion of land use covariates will significantly improve the performance of SDMs and that the differences in the projected distributions produced by climate SDMs and full SDMs will be significantly. By doing so, we provide insights into the conservation of *D. involucrate* in the face of pervasive environmental change.

## 2 MATERIALS AND METHODS

### 2.1 Study area and species occurrence data

**FIGURE 1:**
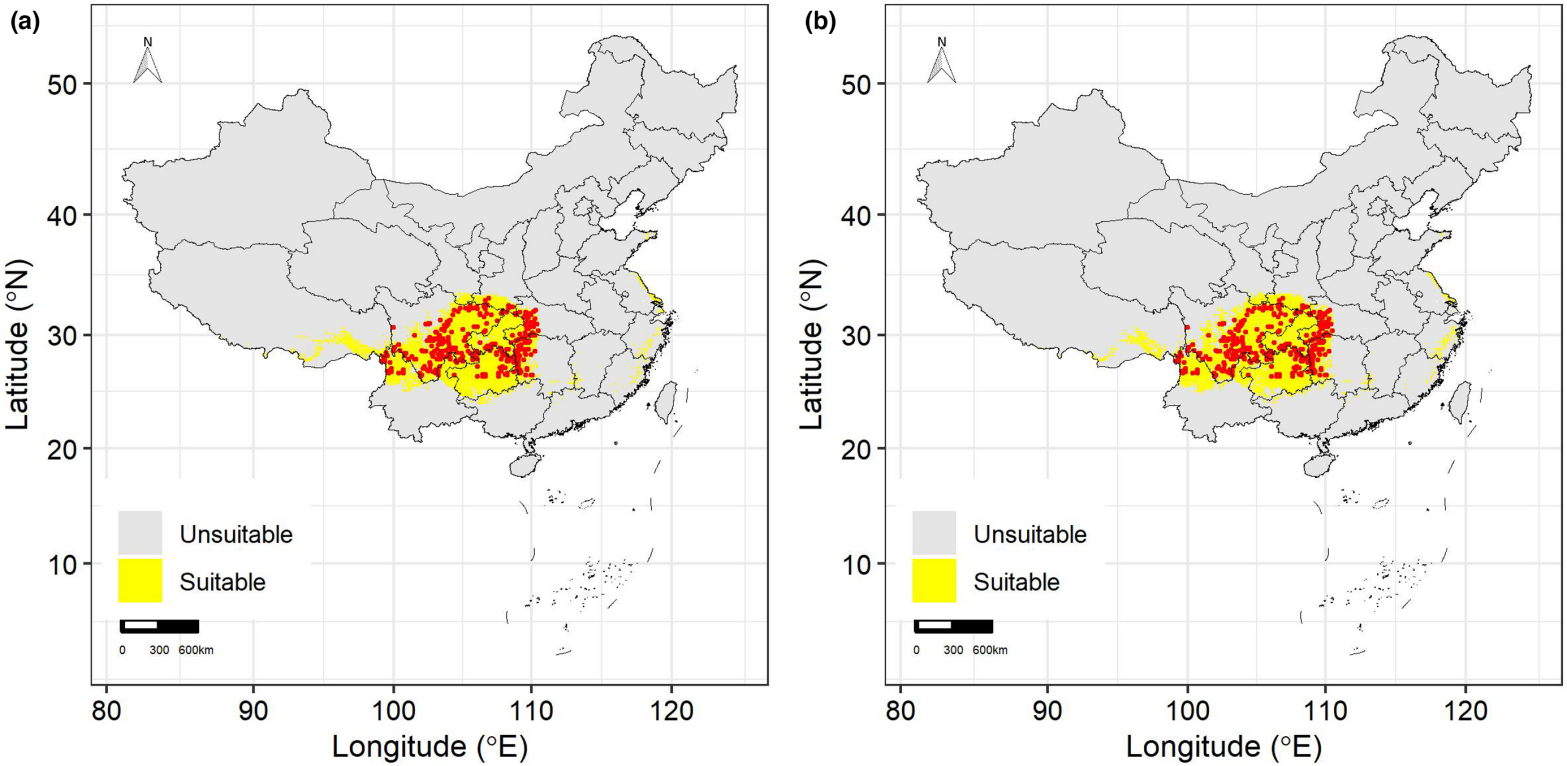
Potential suitable (yellow) and unsuitable (gray) habitat suitability of *Davidia involucrata* Baill. In China projected by climate SDMs (a) and full SDMs (b). Red points represent occurrence records of *D. involucrata*

As *D. involucrate* is a Tertiary relict plant endemic to China, we chose the whole China as the study area following previous studies (Figure 1) (Long et al., 2021b; Tang et al., 2017; Wang et al., 2019). Totally, 337 occurrence records of *D. involucrate* at the spatial resolution of 10 km × 10 km for the time period 1979–2013 were obtained from Long et al. (2021a), in which an extensive database of occurrence records was assembled. Additional details on the methods of collecting and processing the occurrence data can be found in Long et al. (2021b).

### 2.2 Climatic and land use data

Following Long et al. (2021b), we used six bioclimatic variables at a 10 km resolution averaged for the period 1979–2013 from the CHELSA database (Karger et al., 2017) for model training and projection of the current potential distribution of *D. involucrate*: annual mean temperature (BIO1), isothermality (BIO3), temperature annual range (BIO7), precipitation of the driest month (BIO14), precipitation seasonality (BIO15), and precipitation of the warmest quarter (BIO18), for these variables have low multicollinearity (all variables with Pearson correlation coefficients |*r*| < .7 and variance inflation factor VIF < 5) and have the greatest ecological relevance to *D. involucrate* (Long et al., 2021b). Similarly, the same six bioclimatic variables at a 2.5 arc‐min resolution for two future time periods, 2050s (averaged for 2041–2060) and 2070s (averaged for 2061–2080), under two representative concentration pathways (RCPs) scenarios, RCP2.6 and RCP8.5, from six global circulation models (GCMs): CNRM‐CM6‐1, CNRM‐ESM2‐1, CanESM5, IPSL‐CM6A‐LR, MIROC‐ES2L, and MIROC6 were obtained from the WorldClim database (Fick & Hijmans, 2017). To match the resolution of species distribution map, all the bioclimatic variables were resampled at 10 km resolution using a bilinear interpolation.

The current and future land use data at a 1 km resolution were obtained from the FROM‐GLC database (Li et al., 2016), which includes the proportion of ten different land‐use types: (a) bareland, (b) cropland, (c) forest, (d) grassland, (e) impervious, (f) shrubland, (g) snow/ice, (h) urban green spaces, (i) water, and (j) wetland. For consistency with climatic data and avoiding multicollinearity, we extracted six land‐use variables selected with low multicollinearity (i.e., the percentage of area covered by six different land‐use types: cropland, forestland, grassland, shrubland, water, and urban) for three time periods (i.e., 2010 [current], 2050s [average for 2041–2060], and 2070s [average for 2061–2080]) under two RCP scenarios (i.e., RCP 2.6 and RCP 8.5) and masked these land‐use layers to the study area and calculated the proportion of each land‐use type at 10 km resolution.

### 2.3 Species distribution models

To evaluate the effect of climate and land use change on *D. involucrate*, following Jamwal et al. (2021), we computed two different types of SDMs with different sets of variables: (a) including only climate variables (climate SDMs) and (b) adding also land use variables (full SDMs). These SDMs were calibrated with an ensemble forecasting approach under the BIOMOD2 platform version 3.5.1 (Thuiller et al., 2021), using the following ten modeling algorithms: artificial neural network (ANN), classification tree analysis (CTA), flexible discriminant analysis (FDA), generalized additive model (GAM), generalized boosted models (GBM), generalized linear model (GLM), multivariate adaptive regression splines (MARS), maximum entropy (MaxEnt), random forests (RF), and surface range envelope (SRE). For these algorithms require species absence records, we generated 10,000 pseudo‐absence points by randomly sampling without replacement. We then performed cross‐validation on each algorithm, where random subsets of 70% of data were used for model calibration and the remaining 30% for evaluating model performance. At each cross‐validation step, two metrics were used to assess the predictive performance of each algorithm: the area under the relative operating characteristic curve (AUC) and the true skill statistic (TSS). To avoid random bias, this cross‐validation process was repeated 10 times.

To produce robust forecasts of the distribution of *D. involucrate*, we produced an ensemble model based on the algorithms with AUC > 0.80 and TSS > 0.60, which creating a median representation of the predictions of the 10 runs and the selected algorithms together. For each covariate included in the ensemble models, we also estimated variable contribution by calculating the change in correlation between the covariates and the response with and without the selected variable (Thuiller et al., 2021). The final ensemble models were then projected to the environmental conditions under current and future period, respectively. One probability map for habitat suitability was produced for each of the two model types at 2050s and 2070s under 12 future global change scenarios, representing all combinations of the two RCP scenarios and the six GCMs. Finally, all these maps were converted to binary presence absence maps by choosing the probability threshold that maximized the TSS value (Thuiller et al., 2021).

### 2.4 Statistical analysis

Analyses were conducted on the above binary presence/absence habitat suitability maps. To explore the effect of climate and land use change on the projected distribution of *D. involucrate*, following Zhang et al. (2015), we first calculated two metrics of species' vulnerability to future global change: the relative changes in suitable area of *D. involucrate* between current and future periods in the whole study area and in its current suitable range, respectively, following the Equation C = (A − B)/B, where C is the relative change ratio, while A and B are the future and current potential suitable habitat area (km^2^) of *D. involucrate*. The first metric measures to what degree the future suitable area is large or smaller than the current suitable area, while the second metric measures the loss of current suitable habitat (Thuiller et al., 2011, 2014). Second, to examine the distance and direction of spatial shifts, we also took the centroids of the species range from the current and the future periods using the R package “rgeos” with the “gCentroid” function. A positive distance value indicates northerly shift and negative, a southerly shift. Finally, to explore the conservation effectiveness of current nature reserve networks in protecting *D. involucrate* under global change, we obtained spatial polygon data for nature reserve boundaries in China from the World Database on Protected Areas (UNEP‐WCMC, 2017), which was resampled to 1 km spatial resolution. We further calculated the relative changes in potential suitable habitat area inside the current nature reserve networks between current and future periods both in the whole study area and in its current suitable range, respectively.

## 3 RESULTS

### 3.1 Species distribution models

As the AUC and TSS values of the 10 algorithms except SRE are all above 0.8 and 0.7, respectively, we excluded SRE in subsequence analysis (Table 1). Results of paired Wilcoxon signed rank test indicate that full SDMs performed significantly better than climate SDMs in terms of both AUC (*V* = 466, *p* < .001) and TSS (*V* = 835, *p* < .001). Therefore, we hereafter reported the results regarding ensemble models and variable importance only for full SDMs.

**TABLE 1:** Model performance (mean ± *SD*) of ten modeling algorithms (i.e., artificial neural network [ANN], classification tree analysis [CTA], flexible discriminant analysis [FDA], generalized additive model [GAM], generalized boosted models [GBM], generalized linear model [GLM], multivariate adaptive regression splines [MARS], maximum entropy [MaxEnt], random forests [RF], and surface range envelope [SRE]) used for species distribution modeling of *Davidia involucrate* Baill. based on area under curve (AUC) and true skill statistics (TSS) value

Algorithms	AUC		TSS	
	Climate SDMs	Full SDMs	Climate SDMs	Full SDMs
ANN	0.932 ± 0.008	0.951 ± 0.003	0.835 ± 0.017	0.871 ± 0.010
CTA	0.909 ± 0.006	0.920 ± 0.005	0.813 ± 0.008	0.836 ± 0.004
FDA	0.941 ± 0.002	0.956 ± 0.001	0.820 ± 0.003	0.832 ± 0.005
GAM	0.949 ± 0.002	0.951 ± 0.003	0.860 ± 0.005	0.859 ± 0.006
GBM	0.955 ± 0.002	0.960 ± 0.001	0.854 ± 0.005	0.866 ± 0.003
GLM	0.945 ± 0.001	0.954 ± 0.001	0.847 ± 0.004	0.868 ± 0.004
MARS	0.953 ± 0.001	0.961 ± 0.001	0.852 ± 0.005	0.876 ± 0.004
MaxEnt	0.958 ± 0.002	0.962 ± 0.001	0.856 ± 0.006	0.865 ± 0.005
RF	0.960 ± 0.001	0.967 ± 0.001	0.858 ± 0.005	0.869 ± 0.007
SRE	0.830 ± 0.011	0.790 ± 0.007	0.659 ± 0.021	0.579 ± 0.013
Ensemble	0.971	0.978	0.889	0.909

*Note*: The AUC and TSS values of the ensemble species distribution models (SDMs) are also shown for comparison.

**FIGURE 2:**
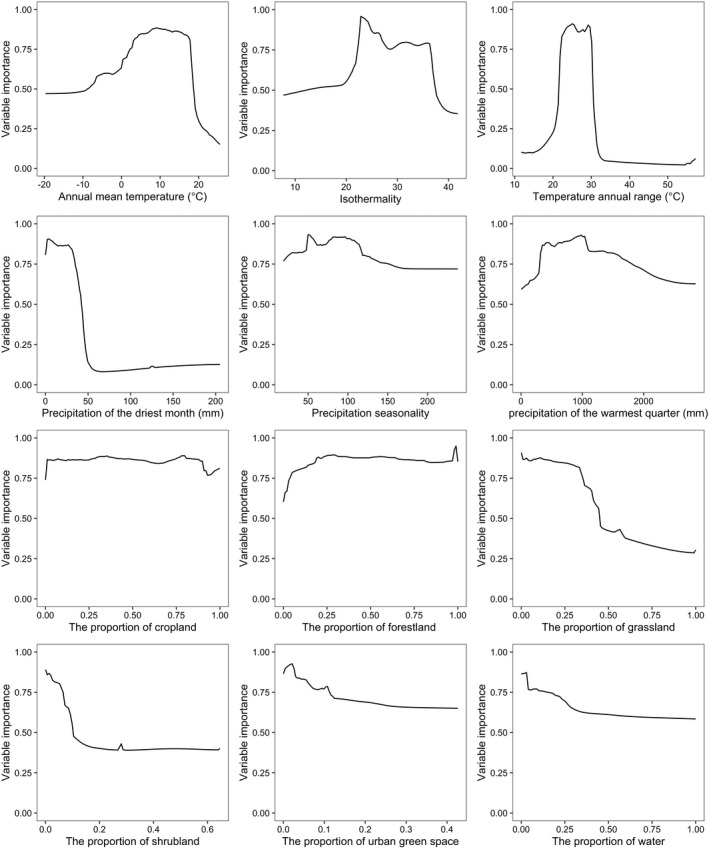
Response curve of environmental variables used to model habitat suitability of *D. involucrate* in China

The AUC and TSS value of the full ensemble model is 0.978 and 0.909, respectively, which is higher than that of the individual algorithms, indicating that our model was statistically robust and the predictive performance was near perfect (Allouche et al., 2006). According to the ensemble model, environmental variables contributed differently to our models (Table 2). The temperature annual range is the most influential variable, contributed 62.0% in the model, followed by annual mean temperature (10.4%), the percentage of grassland cover (9.4%), and precipitation of the driest month (8.1%). Response curves of the above four variables indicate that *D. involucrate* occurs mainly in areas with annual mean temperature ranging from approximately −1.6 to 19.2°C, temperature annual range ranging from approximately 21.3–31.5°C, precipitation of the driest month between about 0.1 and 59 mm, and the proportion of areas covered by grassland <0.59 (Figure 2). The remain eight variables contributed little to the distribution of *D. involucrate* (Table 2).

**TABLE 2:** Relative importance (mean ± *SD*) of the predictor variables in the ensemble model of habitat suitability for *Davidia involucrate*

Predictor variables	Relative importance	
	Climate SDMs	Full SDMs
Annual mean temperature (BIO1)	0.3487 ± 0.0003	0.1321 ± 0.0002
Isothermality (BIO3)	0.0601 ± 0.0007	0.0266 ± 0.0003
Temperature annual range (BIO7)	0.8033 ± 0.0069	0.7856 ± 0.0041
Precipitation of the driest month (BIO14)	0.0997 ± 0.0012	0.1026 ± 0.0009
Precipitation seasonality (BIO15)	0.0154 ± 0.0002	0.0121 ± 0.0003
Precipitation of the warmest quarter (BIO18)	0.0688 ± 0.0008	0.0377 ± 0.0004
The proportion of cropland (CL)	–	0.0019 ± 0.0001
The proportion of forestland (FL)	–	0.0224 ± 0.0002
The proportion of grassland (GL)	–	0.1186 ± 0.0020
The proportion of shrubland (SL)	–	0.0265 ± 0.0005
The proportion of urban green spaces area (UGSL)	–	0.0003 ± 0.0001
The proportion of water (WL)	–	0.0006 ± 0.0001

### 3.2 Projected range shifts under different future scenarios

**FIGURE 3:**
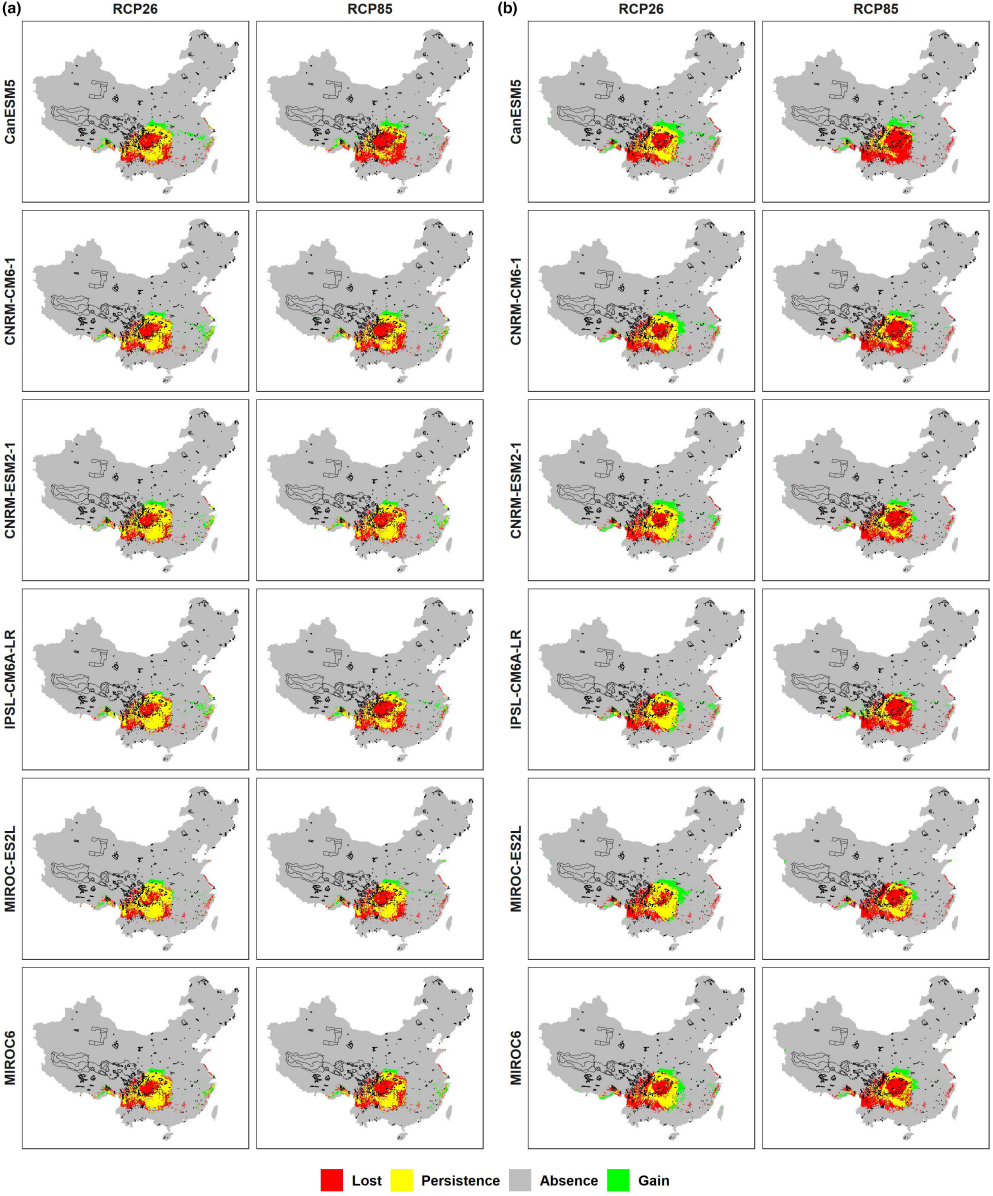
Changes in suitable ranges of *Davidia involucrata* Baill. Projected by full ensemble SDMs under each GCMs and RCP scenario in: (a) 2050s and (b) 2070s. Four trajectories were assigned to each grid cell by comparing habitat suitability under current and future environmental conditions: “Absence,” a grid that is unsuitable for this species under current environmental conditions remain unsuitable under future environmental conditions; “gain,” a grid that is unsuitable for this species under current environmental conditions become suitable under future environmental conditions; “lost,” a grid that is suitable for this species under environmental climatic conditions become unsuitable under future environmental conditions; “persistence,” a grid that is suitable for this species under current environmental conditions remain suitable under future environmental conditions

**FIGURE 4:**
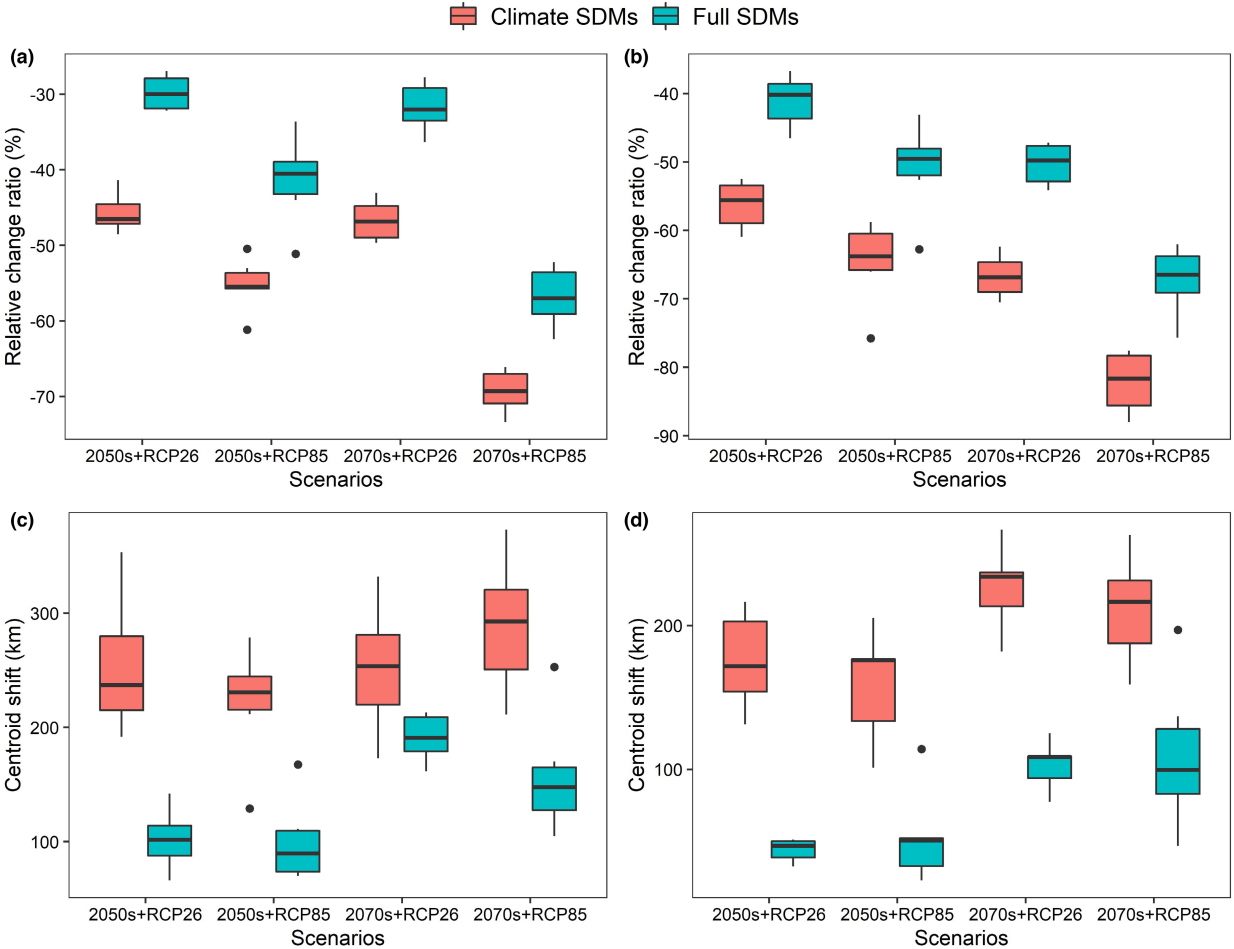
Range shifts of *Davidia involucrate* in 2050s and 2070s under different future scenarios projected by climate SDMs and full SDMs, respectively. (a) The relative change in suitable aera in the whole study area, (b) the relative change in suitable aera in the current suitable range of *D. involucrate*, (c) the distance of centroid shifts in the whole study area, and (d) the distance of centroid shifts in the current suitable range of *D. involucrate*

Overall, both climate SDMs and full SDMs predicted severe range contraction of the future distribution of *D. involucrate*, but full SDMs, by integrating climate and land‐use change scenarios, predicted far less range contraction than climate SDMs, which only involve climate change scenarios (Figures 3 and 4). Specifically, the relative change ratios projected by full SDMs ranged from −51.13% (under CanESM5 and RCP 8.5 scenario) to −26.96% (under CNRM‐ESM2‐1 and RCP 2.6 scenario) by the 2050s and from −62.44% (under CanESM5 and RCP 8.5 scenario) to −27.74% (under CNRM‐ESM2‐1 and RCP 2.6 scenario) by the 2070s in the whole study area. On the contrary, the relative change ratios projected by climate SDMs ranged from −61.16% (under CanESM5 and RCP 8.5 scenario) to −41.36% (under IPSL‐CM6A‐LR and RCP 2.6 scenario) by the 2050s and from −73.38% (under CNRM‐CM6‐1 and RCP 8.5 scenario) to −43.05% (under CNRM‐ESM2‐1 and RCP 2.6 scenario) by the 2070s. Similarly, although the relative change ratios of potential suitable habitats in the current suitable range of *D. involucrate* are greater than those in the whole study area, *D. involucrate* are also more likely to lose range under climate SDMs than full SDMs (Figure 4).

Under all future scenarios, the distance of shift of species distribution centroids is all greater than zero (Figure 4), indicating that *D. involucrate* would likely shift to the northward of high‐latitude regions in response to global change. Furthermore, due to greater magnitude of species' range shifts under future climate conditions, *D. involucrate* was predicted to shift longer distance under climate SDMs than full SDMs (Figure 4). Specifically, the full SDMS predicted that the species would need move from 65.92 km (under the MIROC‐ES2L climate model and RCP 2.6 scenario by the 2050s) to 252.86 km (under the CanESM5 climate model and RCP 8.5 scenario by the 2070s) in the whole study area and from 88.83 km (under the MIROC6 climate model and RCP 8.5 scenario by the 2050s) to 196.96 km (under the CanESM5 climate model and RCP 8.5 scenario by the 2070s) in the current suitable range of *D. involucrate*, while the climate SDMs predicted that the distance of centroid shifts ranged from 101.07 km (under the IPSL‐CM6A‐LR climate model and RCP 8.5 scenario by the 2050s) to 373.02 km (under the CanESM5 climate model and RCP 8.5 scenario by the 2070s) in the whole study area and from 101.07 km (under the IPSL‐CM6A‐LR climate model and RCP 8.5 scenario by the 2050s) to 266.59 km (under the CNRM‐CM6‐1 climate model and RCP 2.6 scenario by the 2070s) in the current suitable range of *D. involucrate*.

### 3.3 The future effectiveness of current PAs

**FIGURE 5:**
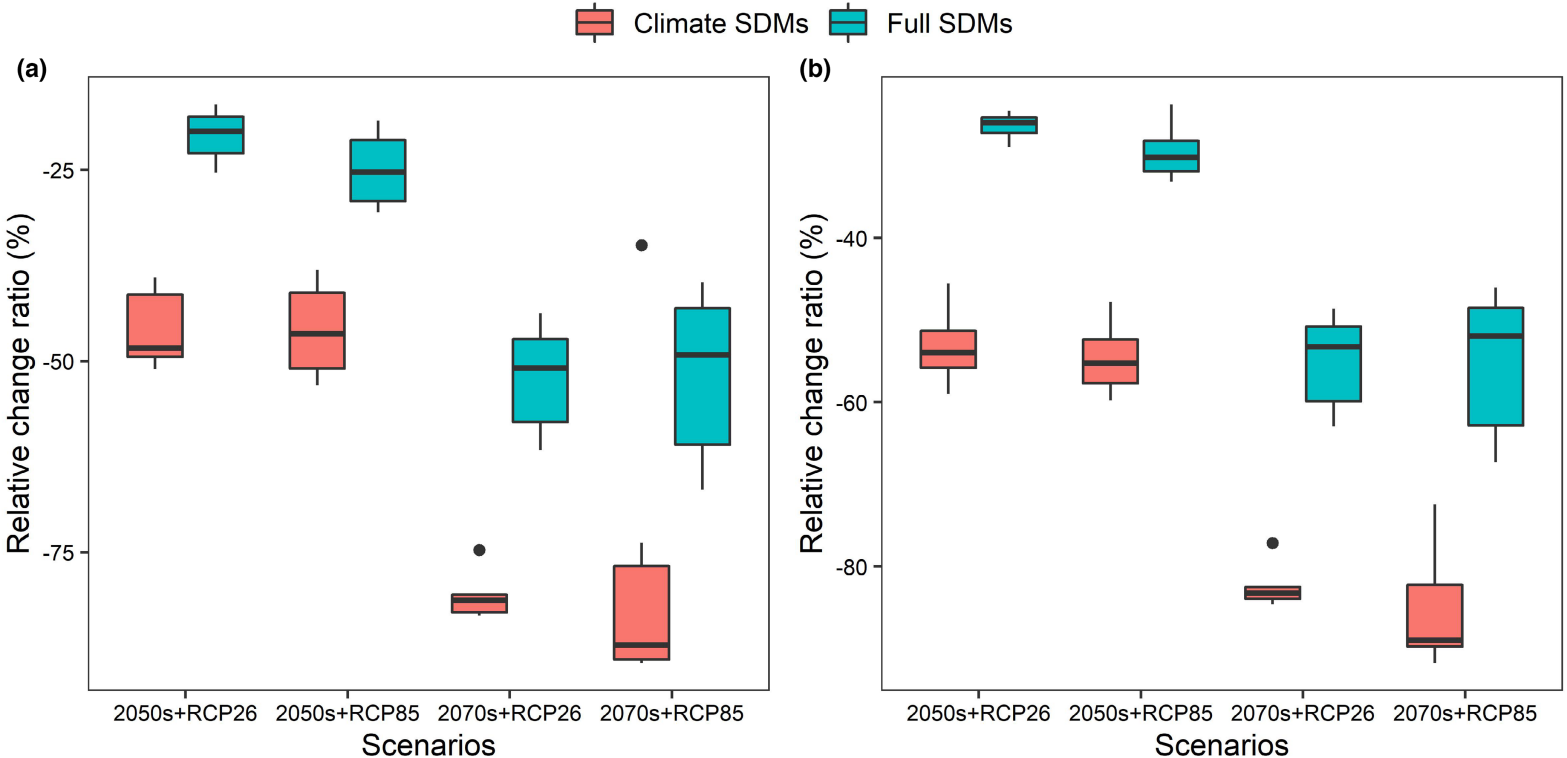
Relative change ratios of the overlap area of current nature reserve networks and potential suitable habitat of *Davidia involucrate* baill. Under different future scenarios projected by climate SDMs and full SDMs, respectively. (a) The relative change ratios of the overlap area in the whole study area, (b) the relative change ratios of the overlap area in the current suitable range of *D. involucrate*

The current PAs only cover 6.8% (61,500 km^2^) of the current suitable habitat of the *D. involucrate*. The relative change ratios of projected suitable habitat inside current PAs have a similar trend in predicted species range size both in the whole study area and in the current suitable range of *D. involucrate* (Figure 5). Especially, *D. involucrate* is also more likely to lose range under climate SDMs than full SDMs inside PAs. Specifically, the relative change ratios inside PAs projected by full SDMs ranged from −66.83% (under MIROC‐ES2L climate model and RCP 8.5 scenario by the 2070s) to −16.42% (under CNRM‐ESM2‐1 climate model and RCP 2.6 scenario by the 2050s) in the whole study area and from −67.32% (under MIROC‐ES2L climate model and RCP 8.5 scenario by the 2070s) to −18.54% (under CNRM‐ESM2‐1 climate model and RCP 8.5 scenario by the 2050s) in the current suitable range of *D. involucrate*. On the contrary, the relative change ratios inside PAs projected by climate SDMs ranged from −89.47% (under CNRM‐CM6‐1 climate model and RCP 8.5 scenario by the 2070s) to −34.85% (under IPSL‐CM6A‐LR climate model and RCP 8.5 scenario by the 2070s) in the whole study area and from −91.73% (under CNRM‐CM6‐1 climate model and RCP 8.5 scenario by the 2070s) to −45.54% (under IPSL‐CM6A‐LR climate model and RCP 2.6 scenario by the 2050s) in the current suitable range of *D. involucrate*.

## 4 DISCUSSION

Accurately predicting the future distribution of species is crucial for understanding how species will response to global environmental change and evaluating the effectiveness of current PAs (Wiens et al., 2009) and has required that projections should cover multiple factors (e.g., climate and land use change) that are important drivers of species distributional changes (Brook et al., 2008; Travis, 2003). In this study, we projected the distribution of suitable habitats of *D. involucrate* under the combination of climate change and land use change scenarios. The results suggest that although *D. involucrate* is extremely vulnerable to future climate change, and future climate change would negate the conservation effectiveness of the current PAs, future land use pattern would offset the negative effects of climate change on the future distributions of *D. involucrate* and the effectiveness of the current PAs. These findings should inform the dialogue determining the roles of land use change on the future distribution patterns of *D. involucrate* and the effectiveness of current PAs, and thus have important implications for the conservation of *D. involucrate*.

To our knowledge, this study is one of the first studies so far to forecast the distributional changes of *D. involucrate* by combining climate change and land use changes. Previous studies have reported that human‐induced land use change are important drivers in determining distribution patterns of *D. involucrate*, for the increasing intensity of human‐induced land‐use change (e.g., logging) has led to a sharp decrease of its remaining habitats (Wang et al., 2019). Consistent with these studies, our findings suggest that *D. involucrate* are influenced by land use variables, among which the most important land use variable is the proportion of the grassland cover. This is partly because its populations are mainly distributed in forests with lower grassland cover compared to other landscape types (He et al., 2004). As expected, both AUC (paired Wilcoxon signed rank test, *V* = 466, *p* < .001) and TSS (*V* = 835, *p* < .001) indicated that incorporating land use variables into climate SDMs greatly improves the model performance, which suggests that climate change alone may over‐represent species range and accordingly lead to unreliable projections of range dynamics (Di Febbraro et al., 2019; Sohl, 2014; Stanton et al., 2012).

Besides, the climate SDMs and full SDMs we implemented allowed us to evaluate the effect of climate and land use change on the future distribution of *D. involucrate*. Previous studies have suggested that climate factors are main drivers in shaping the distribution of *D. involucrate*, for *D. involucrate* is cold intolerance species and mainly distributes in the areas with narrow annual temperature range and high precipitation (Liu et al., 2019, Su & Zhang, 1999). Nonetheless, according to our results, climate SDMs projected less suitable habitats than full SDMs both in the whole study area and in its current suitable range. This finding suggests that including land use variables may project a wider bioclimatic niche and accordingly could create more suitable habitats that species can colonize (Marshall et al., 2018). For example, forest habitats with moderate temperature range emerges as preferred habitats by this species (Wang et al., 2019). Moreover, land use change, such as the abandonment of currently disturbed areas, may offer an opportunity for habitat restoration to preserve the suitable habitats of *D. involucrate* and help them cope with current and future climate change (Navarro & Pereira, 2012; Zamora‐Gutierrez et al., 2018). Together, these findings inform that adopting management actions (e.g., increase forest cover and forbid logging) that contribute to the improvement of the habitats of *D. involucrate* may be an effective way to offset some of the negative effects of a changing climate on *D. involucrate*.

Our results also provide the first assessment of the future effectiveness of the current PAs for the conservation of *D. involucrate* under the combinations of climate and land use change scenarios. Consistent with previous studies (Long et al., 2021b), our results also suggest that future climate would negate the future effectiveness of current PAs for protecting *D. involucrate*, for the species would experience serve range contraction under future climate change inside PAs. However, this effectiveness may be improved when land use variables were incorporated into the climate SDMs, for the percentage of the suitable habitats lost projected by full SDMs were lower than climate SDMs. These results highlight the key role that land use change will play over the next decades to maintain suitable habitats for *D. involucrate* inside PAs.

Overall, this study offers novel insights into how land use change in interaction with climate change might strongly impact on the projected distribution of *D. involucrate*. This study also provides useful information for comprehending the key role that the current PAs will play in the near future by integrating climate and land‐use change scenarios. In the light of our results, we underline the need for an explicit consideration of land use dynamics when forecasting the future distribution of species and the effectiveness of PAs in a context of global change.

## AUTHOR CONTRIBUTIONS

**Junfeng Tang:** Conceptualization (equal); formal analysis (lead); investigation (lead); methodology (equal); project administration (equal); writing – original draft (lead); writing – review and editing (equal). **Xuzhe Zhao:** Conceptualization (equal); funding acquisition (lead); investigation (equal); project administration (lead); resources (lead); supervision (lead); writing – original draft (equal); writing – review and editing (lead).

## CONFLICT OF INTEREST

The authors declare no conflict of interest.

## Data Availability

Datasets used in this study are obtain from Long et al. (2021a), which is available online from the Dryad Digital Repository: https://doi.org/10.5061/dryad.cnp5hqc56.
